# Galangin, a dietary flavonoid, ameliorates hyperglycaemia and lipid abnormalities in rats with streptozotocin-induced hyperglycaemia

**DOI:** 10.1080/13880209.2018.1474931

**Published:** 2018-06-28

**Authors:** Amal A. Aloud, Veeramani Chinnadurai, Chandramohan Govindasamy, Mohammed A. Alsaif, Khalid S. Al-Numair

**Affiliations:** a Department of Food Sciences and Nutrition, College of Food and Agriculture Sciences, King Saud University, Riyadh, Saudi Arabia;; b Department of Community Health Sciences, College of Applied Medical Sciences, King Saud University, Riyadh, Saudi Arabia

**Keywords:** High blood glucose, lipid changes, plasma, tissues, glibenclamide

## Abstract

**Context:** Galangin, a natural flavonoid, is found in honey and *Alpinia officinarum* Hance (Zingiberaceae). Galangin has antiviral, antimicrobial, antidiabetic and anticancer properties, without side effects. The effects of galangin on hyperglycaemia and lipid abnormalities are not known.

**Objective:** To elucidate the effectiveness of galangin on hyperglycaemia-associated complications and lipid changes in rats with streptozotocin (STZ)-induced hyperglycaemia.

**Materials and methods:** Diabetes was induced in adult Wistar rats by administering 40 mg/kg of STZ. In our previous study, galangin had no toxicity at concentrations up to 320 mg/kg. Therefore three doses of galangin (4, 8 or 16 mg/kg BW) or glibenclamide (600 µg/kg BW) were administered daily to diabetic rats orally for 45 days.

**Results:** Diabetic rats showed a significant (*p* < 0.05) increased levels of plasma glucose (281.10 mg/dL) and decreased levels of insulin (6.01 μU/mL). Additionally, diabetic rats showed a significant (*p* < 0.05) increased levels of plasma lipid profiles such as total cholesterol (149.05 mg/dL), triglycerides (143.28 mg/dL), free fatty acids (139.37 mg/dL), phospholipids (127.53 mg/dL), plasma low-density lipoprotein-cholesterol (98.72 mg/dL), plasma very low-density lipoprotein-cholesterol (28.65 mg/dL), and significant (*p* < 0.05) decreased in plasma high-density lipoprotein-cholesterol (21.68 mg/dL). When galangin was administered to the hyperglycaemic rats, plasma glucose and insulin levels and lipid profiles reverted to levels similar to those in healthy control rats.

**Discussion and conclusions:** Administration of galangin reduced hyperlipidaemia related to the risk of diabetic complications and could be beneficial for diabetic hyperlipidaemic patients. Further work detailing its mechanism-of-action for improving hyperglycaemic-associated lipid abnormalities is needed.

## Introduction

Diabetes is a disorder involving multiple complications. Its occurrence has been increasing widely both in developed and developing countries, including Saudi Arabia. According to the World Health Organization (WHO), 285 million people were affected worldwide by diabetes in 2010 and 439 million people are projected to be affected by 2030, especially adults (Shaw et al. 2010). Uncontrolled hyperglycaemia can cause many diabetic complications, including heart, kidney, eye and nervous system diseases. Studies have revealed that lipids, specifically total cholesterol (TC) and triglycerides (TGs), accumulate in chronic hyperglycaemic patients due to deficiencies in insulin action (Biadgo et al. 2017). A profound alteration in TGs and lipids in plasma and lipoprotein profiles in tissues caused by prolonged hyperglycaemia can eventually result in atherosclerosis (Copeland et al. 2018). Therefore, both early diagnosis and appropriate treatment are needed to prevent complications in diabetic patients.

Controlling type 2 diabetes is a global problem and a curative drug has yet to be found. Several medicinal plants and their bioactive ingredients have been assessed in clinical trials for their ability to lower blood glucose levels (Aloud et al. 2017; Shokoohi et al. 2017). The advantages of natural drugs include the absence of toxins and side effects like those often observed for synthetic drugs (Siavash et al. 2017). Recent studies have explored many herbs that possess antihyperglycaemic properties in animals (Kharat et al. 2017; Suresh et al. 2017). In addition, these herbs have potential antioxidant and antihyperlipidaemic properties that can ameliorate diabetes-associated hyperglycaemia (Kharat et al. 2017; Suresh et al. 2017). Studies have shown ﬂflavonoids have antioxidant and antihyperglycaemic properties in diabetic rats (Al-Numair et al. 2015; Aloud et al. 2017).

**Figure 1.**
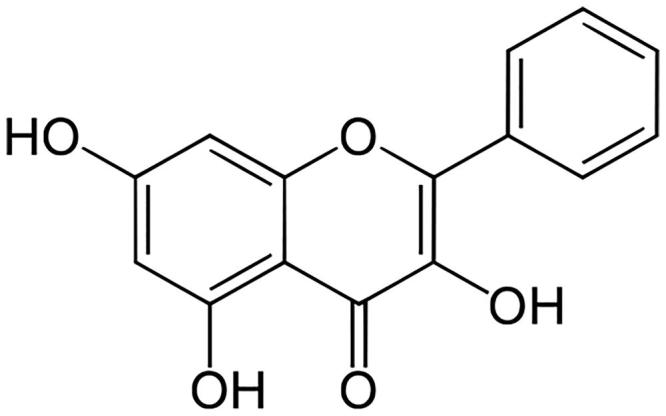
Galangin structure.

Natural flavonoids, which are polyphenolic compounds, have been studied widely as therapeutics for treating cancer (Su et al. 2017), hypertension (Li et al. 2017), hepatotoxicity (Wu et al. 2017) and diabetes mellitus (Al-Numair et al. 2015; Aloud et al. 2017). A natural flavonoid, galangin (3,5,7-trihydroxy-2-phenylchromen-4-one; 3,5,7-trihydroxyflavone) (Figure 1) is found in honey and *Alpinia officinarum* Hance (Zingiberaceae**)** (Lee et al. 2017). Galangin has excellent antimicrobial (Cushnie et al. 2007), antiperoxidative (Aloud et al. 2017), anti-obesity (Kumar and Alagawadi 2013), antitumor (Heo et al. 2001) and anti-inflammatory properties (Jung et al. 2014). Sivakumar and Anuradha (2011) revealed galangin has antioxidative functions in fructose-fed rats. In the present study, an experimental model of diabetes was induced by administering one low dose of STZ to adult male albino Wistar strain rats. Glibenclamide is a standard hypoglycaemic agent that enhances insulin generation from pancreatic β cells. Therefore, glibenclamide was used as a reference drug against which to evaluate the effect of galangin in rats with STZ-induced hyperglycaemia. The effects of galangin on hyperglycaemia and lipid abnormalities have not yet been evaluated. Therefore, we studied the ability of galangin to ameliorate hyperglycaemia-mediated lipid abnormalities in rats with chemically induced hyperglycaemia.

## Materials and methods

### Materials

STZ is a diabetogenic chemical and galangin is a natural compound; both were purchased from Sigma-Aldrich (St. Louis, MO). Galangin purity (CAS Number: 548-83-4) was ≥95% based on high performance liquid chromatography (Sigma-Aldrich, St. Louis, MO). The physicochemical characteristics of galangin are as follows; the empirical formula is C_15_H_10_O_5_, molecular weight is 270.24, melting point is 214–215 °C and dissolving in dimethyl sulphoxide (DMSO). Additional analytical grade chemicals used in these experiments were procured from various commercial suppliers.

### Animals

Nine-week-old male albino Wistar rats (weight: 180–200 g) were bought from the Central Animal House at King Saud University and housed in an air-conditioned room maintained at 25 ± 1 °C with a 12 h light/dark cycle. The animals were fed a normal laboratory pellet diet *ad libitum*. All experiments were conducted in accordance with the Guide for Care and Use of Laboratory Animals, Institute for Laboratory Animal Research, National Institute of Health (NIH Publication No. 80-23; 1996).

### Hyperglycaemia induction using STZ

Hyperglycaemia was induced in healthy animals fasted overnight by intraperitoneally injecting STZ (40 mg/kg BW) that had been dissolved in freshly prepared citrate buffer (0.1 M, pH 4.5). Immediately, 20% glucose was loaded into the drinking water for 24 h after STZ injection to prevent mortality. After 96 h, hyperglycaemia was confirmed in the animals based on glucose measurements. Animals with a plasma glucose level >220 mg/dL were used in subsequent experiments.

### Experimental design for dose fixation

Animals were divided into seven groups, where each group contained six animals. Galangin (4, 8 or 16 mg/kg BW) or glibenclamide (600 µg/kg BW) dissolved in 5% DMSO was administered orally for 45 days. In our previous study, galangin displayed no toxicity for concentrations up to 320 mg/kg (Al-Numair et al. 2015). In this study, three different nontoxic doses of 4, 8 and 16 mg/kg were used. A dose determination study for galangin was performed in STZ-treated, i.e., hyperglycaemic rats.

- *Group 1*: Healthy control rats + 5% DMSO (solvent).
- *Group 2*: Healthy rats + 16 mg/kg BW galangin dissolved in 5% DMSO.
- *Group 3*: Control rats with STZ-induced hyperglycaemia +5% DMSO (solvent).
- *Group 4*: Hyperglycaemic + 4 mg/kg BW galangin dissolved in 5% DMSO.
- *Group 5*: Hyperglycaemic + 8 mg/kg BW galangin dissolved in 5% DMSO.
- *Group 6*: Hyperglycaemic + 16 mg/kg BW galangin dissolved in 5% DMSO.
- *Group 7*: Hyperglycaemic + 600 µg/kg BW glibenclamide dissolved in 5% DMSO.

Animals were sacrificed by cervical dislocation after fasting overnight at the end of day 45 post-STZ administration. Blood was collected to measure plasma glucose and insulin levels. An active dose of galangin was used for further biochemical estimations.

### Experimental design for further various biochemical studies

Animals were assigned into five groups, where each group contained six animals. Rats were treated with galangin (8 mg/kg BW) or glibenclamide (600 µg/kg BW) daily between 9 and 10 am for 45 days.

- *Group 1*: Healthy control rats + 5% DMSO (solvent).
- *Group 2*: Healthy rats + 8 mg/kg BW galangin dissolved in 5% DMSO.
- *Group 3*: Control rats with STZ-induced hyperglycaemia +5% DMSO (solvent).
- *Group 4*: Hyperglycaemic + 8 mg/kg BW galangin dissolved in 5% DMSO.
- *Group 5*: Hyperglycaemic + 600 µg/kg BW glibenclamide dissolved in 5% DMSO.

Animals were sacrificed by cervical dislocation after fasting overnight at the end of experimental day 45. Plasma and tissues were collected and used in further biochemical examinations.

### Biochemical assays

Plasma glucose was analysed using a Trinder reagent kit (Trinder 1969). Plasma insulin was measured by insulin radioimmunoassay kit (Bürgi et al. 1988) (Linco Research, Inc., St. Charles, MO). Total tissue lipid content was determined using a method described by Folch et al. (1957). TC was evaluated by a method described by Allain et al. (1974). High-density lipoprotein-cholesterol (HDL-C) levels were assayed by the Izzo et al. method (Izzo et al. 1981). Low-density lipoprotein-cholesterol (LDL-C) and very low-density lipoprotein-cholesterol (VLDL-C) levels were determined according to the Friedewald et al. method (Friedewald et al. 1972). TG concentrations were analysed using the McGowan et al. method (McGowan et al. 1983). Free fatty acid (FFA) concentrations were determined using the method described by Falholt et al. (1973). Phospholipids (PLs) concentrations were analysed using the Silversmit and Davis method (Silversmit and Davis 1950).

### Statistical analysis

Data variance was analysed by ANOVA (SPSS software package 9.05, SPSS, Chicago, IL) with Duncan's multiple range test. Data are presented as mean of six rats per group ± standard error. *p* < 0.05 was considered statistically significant.

## Results

### Effect of galangin on hyperglycaemia and hyperinsulinaemia

Table 1 presents the effects of galangin on plasma glucose and insulin levels in rats with STZ-induced hyperglycaemia. Hyperglycaemic rats displayed an increase in glucose levels and decrease in insulin levels compared to normal healthy rats. However, treatment of hyperglycaemic rats with galangin restored glucose and insulin to near-normal levels. Three different doses of galangin (4, 8 and 16 mg) were used to treat the hyperglycaemic rats. Of these three doses, 8 mg displayed the maximum improvement in glucose and insulin levels. Therefore, the 8 mg dose was used in further biochemical studies.

**Table 1.** Effect of galangin on plasma glucose and insulin levels in rats with streptozotocin-induced hyperglycaemia.

Groups	Glucose (mg/dL)	Insulin (μU/mL)
Healthy control	89.93 ± 4.74^a^	15.62 ± 1.10^a^
Healthy control + 16 mg galangin	85.11 ± 4.94^a^	15.77 ± 0.91^a^
Diabetic control	281.10 ± 9.57^b^	6.01 ± 0.44^b^
Diabetic + 4 mg galangin	179.86 ± 0.86^c^	8.14 ± 0.79^c^
Diabetic + 8 mg galangin	117.10 ± 11.32^d^	12.08 ± 1.04^d^
Diabetic + 16 mg galangin	140.53 ± 7.24^e^	10.45 ± 1.72^e^
Diabetic + 600 μg glibenclamide	99.49 ± 5.18^f^	14.20 ± 1.55^f^

Data are presented as mean of six rats per group ± standard error. Groups 1 and 2 are not significantly different from each other (a, a; *p* < 0.05). Groups 4, 5, 6 and 7 are significantly different from Group 3 (b vs. c, d, e, f; *p* < 0.05).

### Effect of galangin on plasma lipid profiles

**Figure 2.**
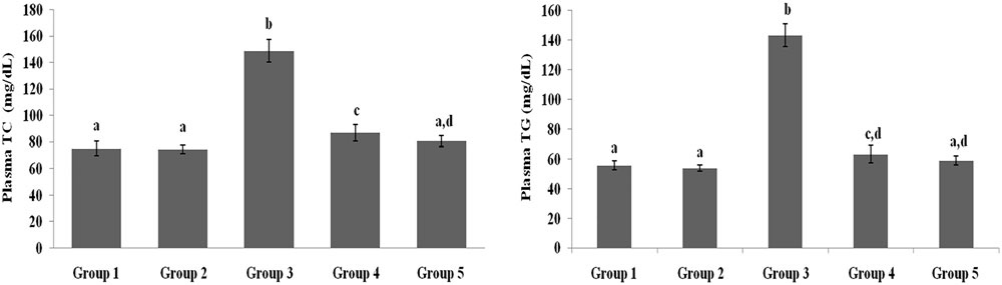
Effect of galangin on abnormal changes in plasma TC and TG levels in rats with streptozotocin-induced hyperglycaemia. Data are presented as mean of six rats per group ± standard error (S.E.). Groups 1 and 2 are not significantly different from each other (a, a; *p* < 0.05). Groups 4 and 5 are significantly different from Group 3 (b vs. c, ad, cd, ad; *p* < 0.05). S.E.: standard error; STZ: streptozotocin. Group 1: healthy control rats; Group 2: healthy control +8 mg galangin; Group 3: diabetic control; Group 4: diabetic +8 mg galangin; Group 5: diabetic +600 µg glibenclamide.

**Figure 3.**
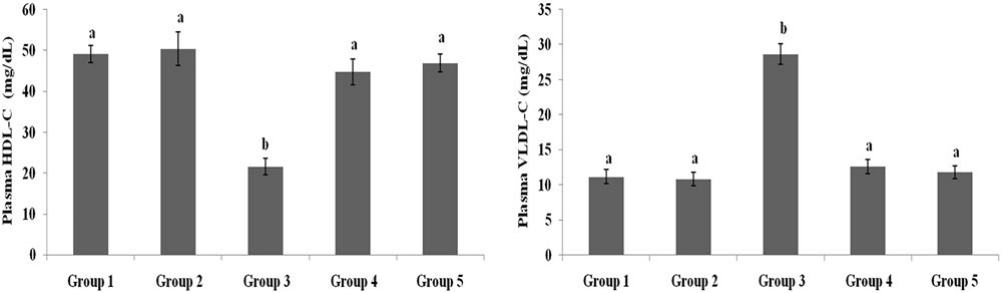
Effect of galangin on abnormal changes in plasma high-density lipoprotein-cholesterol and very-low-density lipoprotein-cholesterol levels in rats with STZ-induced hyperglycaemia. Data are presented as mean of six rats per group ± S.E. Groups 1 and 2 are not significantly different from each other (a, a; *p* < 0.05). Groups 4 and 5 are significantly different from Group 3 (b vs. a; *p* < 0.05). Group 1: healthy control rats; Group 2: healthy control +8 mg galangin; Group 3: diabetic control; Group 4: diabetic +8 mg galangin; Group 5: diabetic +600 µg glibenclamide.

**Figure 4.**
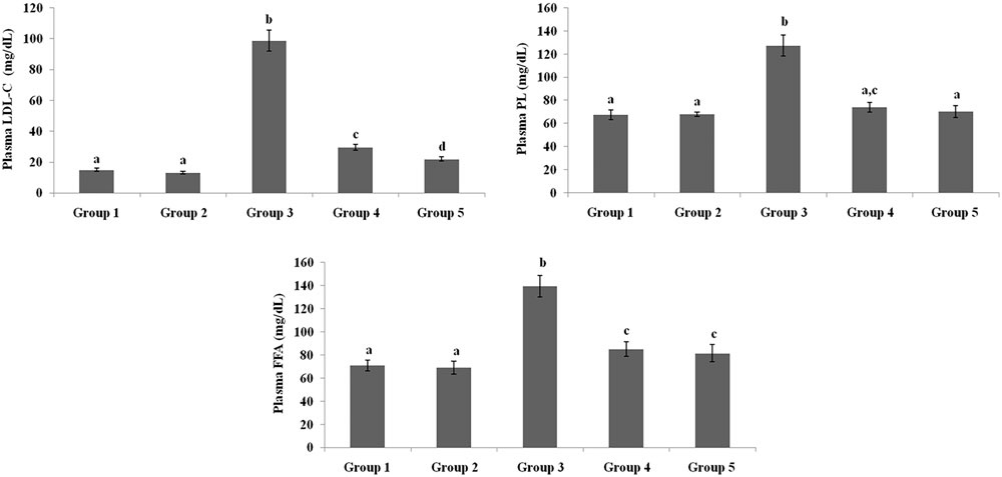
Effect of galangin on abnormal changes in low-density lipoprotein-cholesterol, free fatty acid and phospholipid levels in rats with STZ-induced hyperglycaemia. Data are presented as mean of six rats per group ± S.E. Groups 1 and 2 are not significantly different from each other (a, a; *p* < 0.05). Groups 4 and 5 are significant different compared to Group 3 (b vs. c, d, ac, a; *p* < 0.05). Group 1: healthy control rats; Group 2: healthy control +8 mg galangin; Group 3: diabetic control; Group 4: diabetic +8 mg galangin; Group 5: diabetic +600 µg glibenclamide.

Figures 2–4 present the plasma TC, TG, HDL-C, VLDL-C, LDL-C, FFA and PL levels of healthy and hyperglycaemic rats. The hyperglycaemic rats had increased plasma levels of TC, TG, LDL-C, VLDL-C, FFA and PL and decreased plasma levels of HDL-C. The above lipid abnormalities were restored to near-healthy levels in galangin- and glibenclamide-treated hyperglycaemic rats.

### Effect of galangin on liver and heart lipid profiles

**Figure 5.**
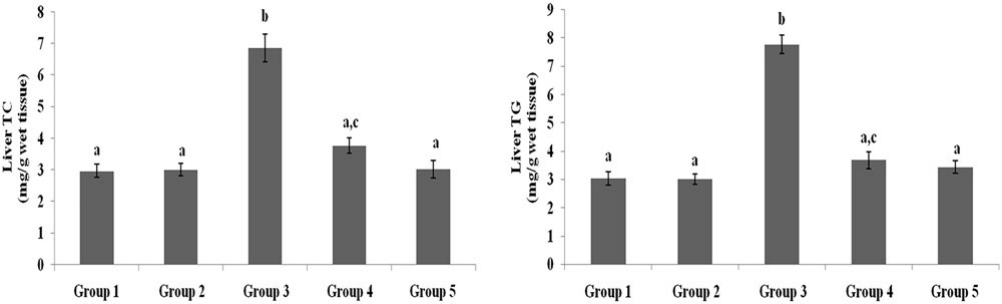
Effect of galangin on abnormal changes in liver total cholesterol and triglyceride levels in rats with STZ-induced hyperglycaemia. Data are presented as mean of six rats per group ± S.E. Groups 1 and 2 are not significantly different from each other (a, a; *p* < 0.05). Groups 4 and 5 are significantly different from Group 3 (b vs. ac, a; *p* < 0.05). Group 1: healthy control rats; Group 2: healthy control +8 mg galangin; Group 3: diabetic control; Group 4: diabetic +8 mg galangin; Group 5: diabetic +600 µg glibenclamide.

**Figure 6.**
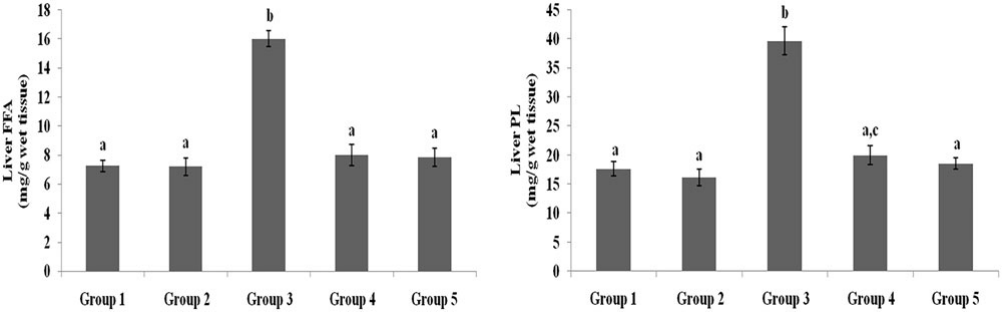
Effect of galangin on abnormal changes in liver free fatty acid and phospholipid levels in rats with STZ-induced hyperglycaemia. Data are presented as mean of six rats per group ± S.E. Groups 1 and 2 are not significantly different from each other (a, a; *p* < 0.05). Groups 4 and 5 are significantly different from Group 3 (b vs. a, ac; *p* < 0.05). Group 1: healthy control rats; Group 2: healthy control +8 mg galangin; Group 3: diabetic control; Group 4: diabetic +8 mg galangin; Group 5: diabetic +600 µg glibenclamide.

**Figure 7.**
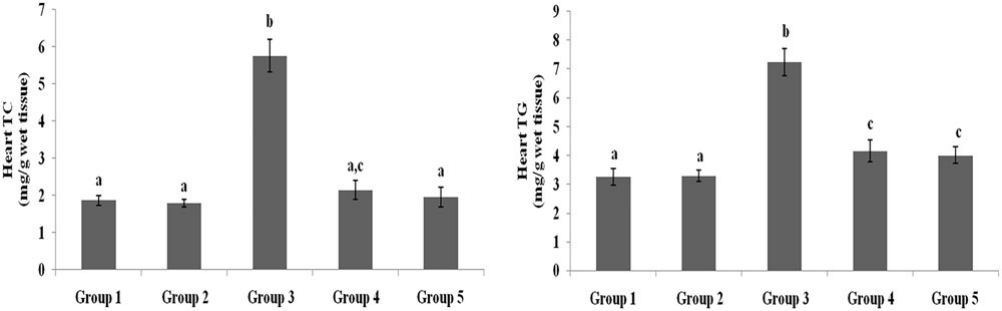
Effect of galangin on abnormal changes of heart total cholesterol and triglyceride levels in rats with STZ-induced hyperglycaemia. Data are presented as mean of six rats per group ± S.E. Groups 1 and 2 are not significantly different from each other (a, a; *p* < 0.05). Groups 4 and 5 are significantly different from Group 3 (b vs. ac, a, c; *p* < 0.05). Group 1: healthy control rats; Group 2: healthy control +8 mg galangin; Group 3: diabetic control; Group 4: diabetic +8 mg galangin; Group 5: diabetic +600 µg glibenclamide.

**Figure 8.**
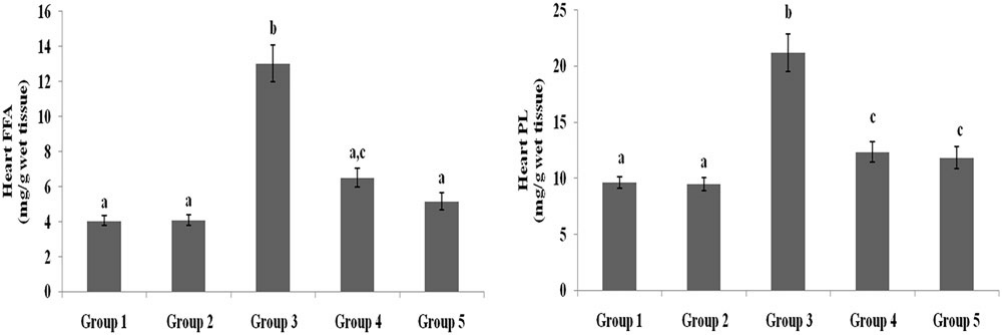
Effect of galangin on abnormal changes in heart free fatty acid and phospholipid levels in rats with STZ-induced hyperglycaemia. Data are presented as mean of six rats per group ± S.E. Groups 1 and 2 are not significantly different from each other (a, a; *p* < 0.05). Groups 4 and 5 are significantly different from Group 3 (b vs. ac, a, c; *p* < 0.05). Group 1: healthy control rats; Group 2: healthy control +8 mg galangin; Group 3: diabetic control; Group 4: diabetic +8 mg galangin; Group 5: diabetic +600 µg glibenclamide.

TC, TG, FFA and PL levels in liver and heart tissues of normal healthy and hyperglycaemic rats are shown in Figures 5–8 and were higher in hyperglycaemic rats than healthy rats. The abnormal lipid levels in tissues of hyperglycaemic rats were restored to levels similar to healthy rats following treatment with galangin and glibenclamide.

## Discussion

STZ impairs insulin activity by selectively damaging pancreatic cells, leading to increased blood glucose levels (Szkudelski 2001). A single low dose of streptozotocin (STZ) enters β cells through the glucose transporter GLUT2 and impairs insulin secretion and action through alkylation of pancreatic β-cell DNA (Szkudelski 2001). Effective improvement of blood glucose levels through enhanced insulin secretion and/or action minimize diabetes-associated risk factors (Turnin et al. 2017). Many natural foods, medicinal plants and plant components have traditionally been used to control diabetes (Al-Numair et al. 2015; Aloud et al. 2017; Suresh et al. 2017; Erfani Majd et al. 2018). Studies have reported that synthetic drugs cause several side effects, including diarrhoea and flatulence (Tai and Fantegrossi 2016). Therefore, researchers have recently begun the search for better agents derived from plants or natural products (Suresh et al. 2017; Erfani Majd et al. 2018). As expected, in a rat model of induced hyperglycaemia, the plasma glucose levels were elevated significantly and insulin levels were decreased significantly compared to healthy rats. Administration of galangin and glibenclamide significantly improved plasma glucose and insulin levels to near-normal. Previous studies have reported flavonoids lower blood glucose levels by activating insulin secretion and/or activity (Al-Numair et al. 2015; Kashchenko et al. 2017). Therefore, the glucose-lowering effect of galangin might be through enhanced insulin secretion and/or action and improved glucose uptake by adipose tissue and muscle.

Chronic hyperglycaemia is a serious metabolic disorder that disturbs carbohydrate and lipid metabolism and is the primary atherosclerotic risk (Wong et al. 2016). Chronic hyperglycaemia is associated with significant changes in plasma TC, TG, FFA and PL levels and cellular membrane lipoprotein profiles that eventually lead to heart disease (Qin et al. 2017; Copeland et al. 2018). A previous study demonstrated dietary and medicinal plant therapies reduce the risk of diabetic complications, especially vascular disease, by influencing plasma lipid profiles (Yamagishi and Matsui 2016). As expected, we noticed increased plasma and tissue TC levels in rats with STZ-induced hyperglycaemia. Upon treatment with galangin, TC levels decreased significantly compared to hyperglycaemic control rats. Normally, circulating LDL-C is taken up in the liver through specific receptors and removed from circulation (Lusis 2000). The present study found increased plasma LDL-C concentrations in hyperglycaemic rats could be due to the failure of LDL-C receptor function. HDL-C is protected by neutralizing oxidized LDL-C, reversing cholesterol transport and inhibiting oxidation of LDL-C. Due to the reciprocal relationship between VLDL-C and HDL-C, increased VLDL-C concentrations may also be responsible for decreased levels of HDL-C. Our study found lower levels of HDL-C, which could be due to reduced lecithin cholesterol acyl transferase enzyme activity. We also observed an increase in HDL-C levels and decrease in LDL-C and VLDL-C levels in galangin-treated hyperglycaemic rats, confirming the effectiveness of galangin in controlling diabetes-related complications.

An increase in blood TG levels is a common problem in hyperglycaemic patients and plays a role in vascular complications (Naqvi et al. 2017). A previous study demonstrated defective lipoprotein lipase (LPL) activity may be responsible for hypertriglyceridemia in diabetics (Trent et al. 2014). In the present study, plasma and tissue TG levels increased significantly in diabetic rats, which might be due to defective LPL. Insulin plays an important role inhibiting hormone-sensitive lipase. In addition, glucagon and other hormones stimulate lipolysis. Therefore, higher serum lipid levels in patients with diabetes could be due to the lack of inhibition of lipolytic hormone activity on the depots (Trent et al. 2014). Antidiabetic drugs are associated with lowered plasma TG due to returning LPL to normal activity (Liu et al. 2018). Treatment with galangin led to decreased TG levels, which may be due to increased insulin secretion as a result of increased LPL activity.

PLs are susceptible substrates for oxygen and hydroxyl free radicals and play important roles in biomembranes, especially maintenance of cellular integrity, microviscosity and survival. In the present study, plasma and tissue PL levels were significantly increased in hyperglycaemic rats, but were significantly decreased to levels similar to those in healthy rats following galangin treatment. FFAs are a crucial source of energy in tissues and act as oxidative fuel for the liver, renal cortex, myocardium and resting skeletal muscle (Shigiyama et al. 2017). Elevated FFA levels have been observed in patients with insulin-resistant diabetes (Ma et al. 2017). Studies have reported that elevated levels of plasma FFA may be associated with the pathophysiology of diabetes through activation of insulin resistance (Bergman and Ader 2000; Shigiyama et al. 2017). Patients with diabetes have shown enhanced blood lipid profiles due to increased mobilization of FFAs from fat depots (Amor et al. 2016). Moreover, a study demonstrated that higher FFA levels are a cardiovascular risk factor in patients with diabetes. In the present study, the plasma and tissue FFA levels increased significantly in hyperglycaemic rats. Upon treatment with galangin, FFA levels decreased significantly to levels near those in healthy rats, which could be due to increased insulin secretion, as well as inhibition of hormone-sensitive lipase activity.

Flavonoids are considered safe to use in the treatment of diabetes-associated lipid abnormalities, as well as other metabolic diseases. Studies have demonstrated flavonoids have a promising role preventing obesity-related diabetic complications due to their effectiveness increasing insulin secretion, reducing insulin resistance, and inhibiting hormone-sensitive lipase activity (Obafemi et al. 2017; Liu et al. 2018). The influence of flavonoid-galangin on lipid profiles in STZ-induced rats could be due to enhanced insulin secretion and/or action through enhancing adipose tissue and muscle glucose uptake, as well as inhibiting hormone-sensitive lipase activity.

We conclude that galangin has significant antihyperlipidaemic and antidiabetic properties. However, the mechanisms-of-action underlying the ability of galangin to improve hyperglycaemic-associated lipid abnormalities remain to be studied in the future.
